# Exploring molecular signatures in PURA syndrome using muscle proteomics and serum biomarkers

**DOI:** 10.1007/s00415-026-13621-7

**Published:** 2026-01-23

**Authors:** Magdalena Mroczek, Corinna Preusse, Andreas Hentschel, Magdalena Chrościńska-Krawczyk, Michał Bielak, Adela Sobolewska, Adela Della Marina, Anisa Hila, Stanley Iyadurai, Florian Kraft, Venkatesh Kumar Chetty, David Muhmann, Tobias Ruck, Hans-Hilmar Goebel, Ulrike Schara-Schmidt, Vera Dobelmann, Basant Kumar Thakur, Werner Stenzel, Andreas Roos

**Affiliations:** 1 Department of Biomedicine, University Hospital Basel, University of Basel, Basel, Switzerland; 2 Department of Consultation-Liaison-Psychiatry and Psychosomatic Medicine, University Hospital Zurich, University of Zurich, Zurich, Switzerland; 3 Department of Neuropathology, Charité – Universitätsmedizin Berlin, Corporate member of Freie Universität Berlin and Humboldt-Universität Zu Berlin, Berlin, Germany; 4 Department of Neurology With Experimental Neurology, Charité – Universitätsmedizin Berlin, Corporate member of Freie Universität Berlin and Humboldt Universität Zu Berlin, Berlin, Germany; 5 Department of Neuropaediatrics, Charité – Universitätsmedizin Berlin, Corporate member of Freie Universität Berlin and Humboldt-Universität Zu Berlin, Berlin, Germany; 6 Leibniz-Institut Für Analytische Wissenschaften -ISAS- E.V., Dortmund, Germany; 7 Department of Child Neurology, University Children Hospital, Lublin, Poland; 8 Department of Pediatric Neurology / Centre for Neuromuscular Disorders in Children, University Duisburg-Essen, Hufelandstrasse 55, 45122 Essen, Germany; 9 Department of General, Visceral, Vascular and Transplant Surgery & Department of Gastroenterology, Hepatology and Transplant Medicine, University Hospital Essen, Essen, Germany; 10 Division of Neurology, Johns Hopkins All Children’s Hospital, St. Petersburg, FL 33701 USA; 11 Institute for Human Genetics and Genomic Medicine, Medical Faculty, RWTH Aachen University, Aachen, Germany; 12 Department of Neurology with Heimer Institute for Muscle Research, University Hospital Bergmannsheil, Bochum, Germany

**Keywords:** Congenital myasthenic syndrome (CMS), Muscle proteomics, Extracellular vesicles in neuromuscular diseases, Thrombospondin-4 (TSP4), Periostin (POSTN), Target of Nesh-SH3 (TARSH)

## Abstract

**Background and purpose:**

Dominant *PURA* variants (encoding purine-rich element-binding protein A) cause a neurodevelopmental disorder with hypotonia, cognitive impairment, and variable neuromuscular symptoms. Clinical presentations and response to pyridostigmine, moreover, highlighted neuromuscular junction (NMJ) involvement. However, NMJ architecture, underlying molecular mechanisms, and potential minimally invasive biomarkers in PURA syndrome remain poorly characterized. This study aimed to profile PURA-related disease using integrated clinical, histological, ultrastructural, transcriptional, and protein analyses of skeletal muscle and blood.

**Methods:**

Ten genetically confirmed patients underwent detailed phenotyping with emphasis on congenital myasthenic syndrome (CMS)-like features. Quadriceps biopsy from one patient was analyzed by histology, immunohistochemistry, and electron microscopy. Protein profiling of muscle, serum, and extracellular vesicles (EVs) was performed by ELISA and mass spectrometry, with validation by qPCR.

**Results:**

In line with the recognized classification of PURA syndrome as a CMS subtype, our patients exhibited hypotonia, ptosis, ocular weakness, and myopathic facies, reflecting impaired neuromuscular transmission. Subtle vesicle accumulation and minor NMJ alterations suggest possible neuromuscular involvement in PURA syndrome. Muscle proteomics showed reduced PURA protein and dysregulation of transcriptional regulation, vesicle transport, extracellular matrix remodeling, and complement activation. qPCR confirmed *POSTN* and *PHGDH* upregulation among others. Serum analyses demonstrated elevated TSP4, identifying a promising candidate blood biomarker for *PURA*-associated NMJ dysfunction. EV proteomics revealed dysregulated immunoglobulins, complement components, and novel candidates including NOTCH2, TARSH, and PON1.

**Conclusions:**

Pathogenic *PURA* variants may impair NMJ structure and vesicle homeostasis, potentially linking molecular and ultrastructural defects with clinical myasthenic features and pyridostigmine responsiveness. Proteomic analysis of skeletal muscle provides initial molecular insights into the consequences of dominant PURA variants in muscle tissue. The identification of TSP4 and extracellular vesicle-associated proteins as potential minimally invasive biomarkers provides a framework for biochemical monitoring of PURA syndrome.

**Supplementary Information:**

The online version contains supplementary material available at 10.1007/s00415-026-13621-7.

## Background

*PURA*-related neurodevelopmental disorders (*PURA*-NDDs) are rare genetic-based phenotypes with more than 700 cases registered globally in the PURA register (as of 2025) [1] and over 600 individuals reported on ClinVar3 or in the literature, but the detailed clinical phenotype and genotype–phenotype correlations have been delineated for a relatively small number of cases (*n* = 54) [2]. However, clinical manifestation is associated with the presence of pathogenic autosomal dominant de novo point mutations within the *PURA* gene or, rarely, heterozygous deletions encompassing the *PURA* gene [1]. However, it is speculated that the real number of PURA patients is higher than reported, given that not all patients are registered and phenotypes may also present as very mild; recently, a patient and his mother with a very mild phenotype presenting as borderline-low IQ and mild dysarthria were reported [2].

*PURA*-NDD was primarily classified as a central nervous system syndrome characterized by neurodevelopmental delay, abnormal speech (often the patients can speak a few words only or do not speak at all), and lack of or impaired ambulation with ataxic gait [3]. Other features include central hypotonia, recurrent apneas, hypersomnolence, feeding difficulties, disconjugate eye movements, and visual abnormalities. PURA patients may also suffer from epileptic, both motor and non-motor seizures [3, 4]. Of note, recently additional phenotypic features, such as fluctuating weakness, myopathic face, ptosis, and fluctuating deep tendon reflexes were described suggesting a multi-systemic origin with a muscular or more precisely a neuromuscular junction defect [5]. Currently, NMJ dysfunction is defined by electrophysiological evidence (e.g., decrement on repetitive nerve stimulation or jitter on single-fiber EMG) and/or by a clinical response to agents enhancing neuromuscular transmission, such as pyridostigmine. Thus, the phenotypic expansion, in accordance with data available from electrophysiological studies showing pathological decrements and results from therapeutic interventions showing beneficial effects [6, 7], led to the re-classification of the PURA syndrome as a *PURA*-CMS [8, 9].

PURA (MIM: 616,158), a member of the PUR protein family (PURα/PURA, encoded at chromosome band 5q31, PURβ/PURB, at 7p13, and two isoforms of PURγ/PURG, at 8p11), is ubiquitously expressed and plays pleiotropic roles across cellular processes. It exhibits the ability to bind to proteins in addition to single-stranded DNA and RNA, thereby influencing DNA replication as well as RNA translation [10]. Among the DNA-binding targets implicated in muscle and nervous system function are *Acta2*, *Apbb1* [11], and *Mbp* [11]. RNA targets include CGG repeats [12, 13] circSamD4 [14], and C9orf72 repeat expansions [15]. CircSamD4, a conserved circular RNA, sequesters PUR proteins to relieve repression of Mhc transcription. Its overexpression promotes myogenesis, while silencing impairs differentiation and reduces myogenic markers in mouse and human cells [14]. Confirmed protein interaction partners of PURA include PURB [16], FUS [17], FMR1 [18], hnRNPU [16], KIF5 [16], MYO5A [19], and STAU1 [16, 20]. In addition, PURA and PURB regulate genes encoding alpha- and beta-myosin heavy chains, key sarcomeric proteins in skeletal muscle [20–22].

In the central nervous system (CNS), PURA is highly expressed and localizes to paraspeckles (membraneless nuclear compartments). One of its RNA partners, *BC1*, is involved in synaptic transmission; its absence is linked to altered glutamatergic signaling and behavioral changes [23]. PURA co-localizes with the dendritic marker MAP2, and its absence in knockout mice leads to a marked reduction in MAP2 staining in both the hippocampus and cerebellum.

PURA co-localizes with STAU1 in dendritic branches implicated in dendrite formation and with FMRP at synapses [24]. In the latter scenario, PURA and FMRP co-localize in multiple small dendritic foci. However, the significance of this interaction in synaptic spine regulation remains to be clarified, especially in the context of PURA syndrome, where dysregulated synaptic pruning and cellular proliferation are observed [18]. These results underscore the functional involvement of PURA in synapses, including the neuromuscular junction (NMJ). This postulate is further supported morphological results from a mouse model of Pura deficiency, which shows that mice exhibit a ~ 70% reduction in synapse formation in the hippocampus [25].

Although the function of the PURA protein in the central nervous system is well reported, its molecular impact to muscle pathologies including impaired neuromuscular transmission remains limited, and related insights into pathobiochemical processes as well as biomarkers are still lacking. To address this gap of knowledge, we clinically characterized a cohort of 10 *PURA* patients. We performed ELISA-based studies of TSP4, a secretory glycoprotein that is localized at NMJs [26] and thus potentially linked to perturbed neuromuscular transmission. In addition, proteomic profiling and electron microcopy were conducted on a muscle biopsy specimen derived from one of these patients. Moreover, we investigated the proteomic signature of extracellular vesicles isolated from serum derived from *PURA* patients. Our combined findings identified TSP4 as the first PURA-related blood biomarker and unveiled subtle ultrastructural changes of skeletal muscle including the NMJ. Dysregulated proteins identified in skeletal muscle are more likely to reflect NMJ dysfunction, although they may also represent non-adaptive changes due to direct muscle cell vulnerability. In contrast, proteins dysregulated in serum, including extracellular vesicles, are more likely to capture broader cellular dysfunction associated with PURA syndrome.

## Materials and methods

### Patient cohort

Patients were included based solely on the presence of a genetically confirmed *PURA* variant. No electrophysiological data were available, and NMJ involvement was not an inclusion criterion. All patients were evaluated in specialized neuromuscular clinics, allowing comprehensive clinical assessment. Along this line, the ten patients included were clinically characterized including a screening for features known to be associated with congenital myasthenic syndromes (CMS) [27]. Eight of these patients were seen by a pediatric neurologist at the University Children´s Hospital, Department of Child Neurology, Lublin, Poland, between 2022 and 2025 and two of them between 2024 and 2025 at the University Duisburg-Essen, Department of Pediatric Neurology, Centre for Neuromuscular Disorders, Essen, Germany. Blood was collected from all patients for ELISA-based biomarker studies (see below). A muscle biopsy was collected from one patient for diagnostic workup prior to molecular genetic diagnosis being available. All parents/caregivers of the patients agreed on participation in the study (including utilization of photographs) and provided written consent. Study approval was obtained from the University Duisburg-Essen ethics committee (approval number 19–9011-BO).

### Microscopic studies on a *quadriceps* muscle biopsy tissue of a PURA patient

Myopathological findings (on the histological, enzyme histochemical and immunohistological, as well as ultrastructural level) were obtained from one patient, later genetically diagnosed with PURA syndrome (P6). These investigations included H&E, Gömöri-trichrome, NADH, ATPase 4.3, ATPase 4.6, ATPase 9.3, COX-SDH, acid phosphatase, and CD56.

For electron microscopic studies, skeletal muscle tissue was fixed in 2.5% glutaraldehyde in 0.1 M sodium cacodylate buffer for 48 h at 4 °C. Samples were post-fixed in 1% osmium tetroxide in 0.05 M sodium cacodylate buffer for 3 h, dehydrated in a graded acetone series including “en bloc” staining with 1% uranyl acetate and 0.1% phosphotungstic acid in the 70% acetone step for 60 min and then embedded in araldite resin. Ultrathin sections were imaged with a Zeiss P902 electron microscope.

### Proteomics on a *quadriceps* muscle biopsy tissue of a PURA patient

The proteomic signature was studied on whole protein extracts from skeletal muscle tissue in a data-independent acquisition (DIA) mode as described before [28].

### Targeted quantitative PCR studies to validate proteomic findings

In addition to the above-mentioned immunostaining studies, qPCR studies were conducted to confirm that altered protein dysregulations are caused by dysregulated gene transcription. Total RNA was extracted from whole muscle tissue via Trizol/chloroform and cDNA was synthesized using the High-Capacity cDNA Archive Kit (Applied Biosystems, Foster City, CA). For qPCR reactions, 20 ng of cDNA were used. For subsequent analysis, the QuantStudio 6 Flex System (Applied Biosystems, Foster City, CA) was utilized with the following running conditions: 95 °C 0:20, 95 °C 0:01, 60 °C 0:20, 45 cycles (values above 40 cycles were defined as not expressed). All targeted transcripts were investigated as technical triplicates. For each of these runs, the reference gene was included as an internal control to normalize the relative expression of the targeted transcripts. Along this line, 16 different transcripts were targeted as summarized in Supplementary Table 2.

### Collection of serum samples

Whole blood from PURA patients was collected using serum monovette tubes. The samples were left undisturbed at room temperature for 15 min to allow clot formation. Serum was separated by centrifugation at 3800 × g for 10 min at 10 °C. The resulting supernatant (serum) was carefully pipetted off and aliquoted into 1.5 ml low-binding Safe-Lock Eppendorf tubes (Eppendorf AG, Hamburg, Germany). Aliquots were immediately stored at −80 °C until further use.

### Enzyme-linked immunosorbent assay (ELISA) studies to investigate thrombospondin-4 (TSP4) level in serum of *PURA patient*s

TSP4, a protein playing a crucial role at the NMJ, was recently described by us as a serum biomarker in pediatric 5q-SMA patients [29]. We hypothesized that TSP4 might also represent a minimal-invasive biomarker of pathophysiological relevance in *PURA* patients. To address this assumption, ELISA-based quantification was carried out on serum samples derived from ten genetically confirmed *PURA* patients as described previously [29]. In addition, ten normal controls (serum derived from age-matched healthy individuals) were included. Serum TSP4 concentrations were measured using a commercial sandwich ELISA kit (Assay Genie, HUES02731) according to the manufacturer’s instructions. Briefly, serum samples were allowed to clot at room temperature for 2 h at 4 °C, followed by centrifugation at 1000 × *g* for 15 min at 4 °C. Samples were diluted 1:10 in the provided sample diluent prior to analysis. Standards and diluted samples (100 µL) were added in duplicate to wells pre-coated with a TSP4-specific capture antibody and incubated for 90 min at 37 °C. Following incubation, wells were treated sequentially with biotinylated detection antibody and HRP conjugate, with appropriate washing steps between incubations. Substrate solution was added for color development, and the reaction was stopped with stop solution. Optical density was measured at 450 nm, and TSP4 concentrations were calculated from a standard curve, accounting for the dilution factor.

### Study of proteomic signature of extracellular vesicles purified from serum of PURA patients

Prompted by the ultrastructural and proteomic findings indicative of altered vesicle homeostasis in *PURA*-mutant muscle, we moreover studied the protein composition of small extracellular vesicles (sEVs, hereafter referred to as EV) isolated from sera derived from *PURA* patients (*n* = 10) and controls (*n* = 5). In brief, EVs were isolated from the serum samples using size exclusion chromatography (SEC). A 500 μl volume of serum was loaded onto a Gen2 qEVoriginal 35 nm SEC column (IZON Science, Christchurch, New Zealand), and EV-containing fractions (F2, F3, and F4; 500 μl each) were collected according to the manufacturer’s instructions. Proteomic profiling was carried out as described before [30].

## Results

### Clinical findings in a cohort of ten PURA patients

**Fig. 1:**
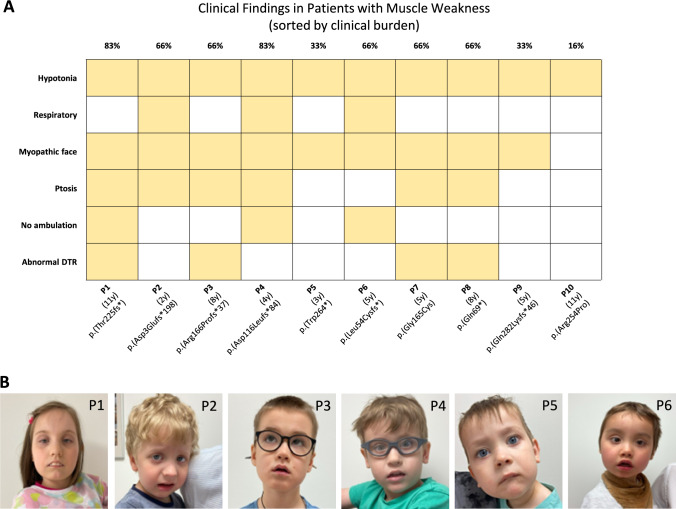
*Clinical findings resembling CMS features in PURA patients* **A** Heatmap showing the presence (yellow) or absence (white) of six clinical features across the ten patients (P1–P10). These features were selected based on their known association with PURA syndrome and potential NMJ involvement. The first line of labels shows the patient code (P1–P10), the second line shows the age at examination in years, and the third line shows the genetic variant (protein change). Clinical features are listed on the y-axis: hypotonia, respiratory involvement, myopathic face, ptosis, no ambulation, and abnormal deep tendon reflexes (DTR). Hereby, respiratory involvement refers to sleep-related apneas without chronic respiratory insufficiency or ventilator dependence, and abnormal DTR indicates variable deep tendon reflex abnormalities (hyper- or hyporeflexia, limb dependent). **B** Photographs of six representative patients (P1–6) showing symptoms related to the neuromuscular weakness in PURA-CMS such as ptosis (P1 and P6), facial hypomimia with opened mouth (P1, P2, P3, P4, and P6), and strabismus (P1 and P6)

The patients of our cohort all exhibited different *PURA* variants (Fig. 1A) and showed the general clinical features of the PURA syndrome, including severe intellectual disability (100%) and neonate-onset symptoms such as hypotonia (100%), respiratory problems (37%), feeding difficulties (37%), hypersomnolence (25%) and ophthalmological problems (87%) [3]. Formal cognitive testing using the WISC-V was performed in one patient (patient 10 at the age of 10 years, 8 months), revealing a full-scale IQ of 60. No patient was diagnosed with epilepsy. Drooling was present to varying degrees in nearly all patients. One patient had severe spine scoliosis and will require surgery, and one patient had a hip dislocation at birth and already had undergone surgery. One patient presented with mild scoliosis. CMS-like features, including ptosis and myopathic facies, were observed in several patients. Oculomotor abnormalities were characterized by complex central disturbances (including nystagmus and impaired gaze control), rather than isolated ophthalmoparesis, consistent with previous reports in PURA syndrome (Fig. 1A, B). Most patients exhibited central apneic events; early respiratory testing was not performed in all cases. No child required nocturnal non-invasive ventilation or tracheostomy, and recurrent respiratory infections were generally not observed. Hypersomnia was pronounced in some patients, most notably P8, who slept 21 h/day until age 2–3 and approximately 18 h/day at age 7. P1 also showed hypersomnia during the first year of life, which resolved after age 2–3 years. Based on the medical history reported by parents, hypotonia, apnea, and eating tended to improve with age, although this was not investigated systematically. The features that may be related to the neuromuscular/NMJ impairment are summarized in Fig. 1A. While these clinical features are suggestive of neuromuscular transmission impairment, we do not claim that NMJ involvement occurs in all *PURA* patients.

The boy (patient 6; P6), who underwent a muscle biopsy for diagnostic purposes, presented as a generalized floppy infant with marked hypotonia in the neck and upper limb muscles. Over time, upper extremity strength improved, whereas neck muscle strength remained unchanged. He moreover presented mild ptosis, horizontal nystagmus, hypersomnia, and apnea with low O_2_ saturation and CO_2_ retention. He avoided eye contact and had poor sucking reflexes and a weak cry.

### Morphological findings in skeletal muscle of a PURA patient

**Fig. 2:**
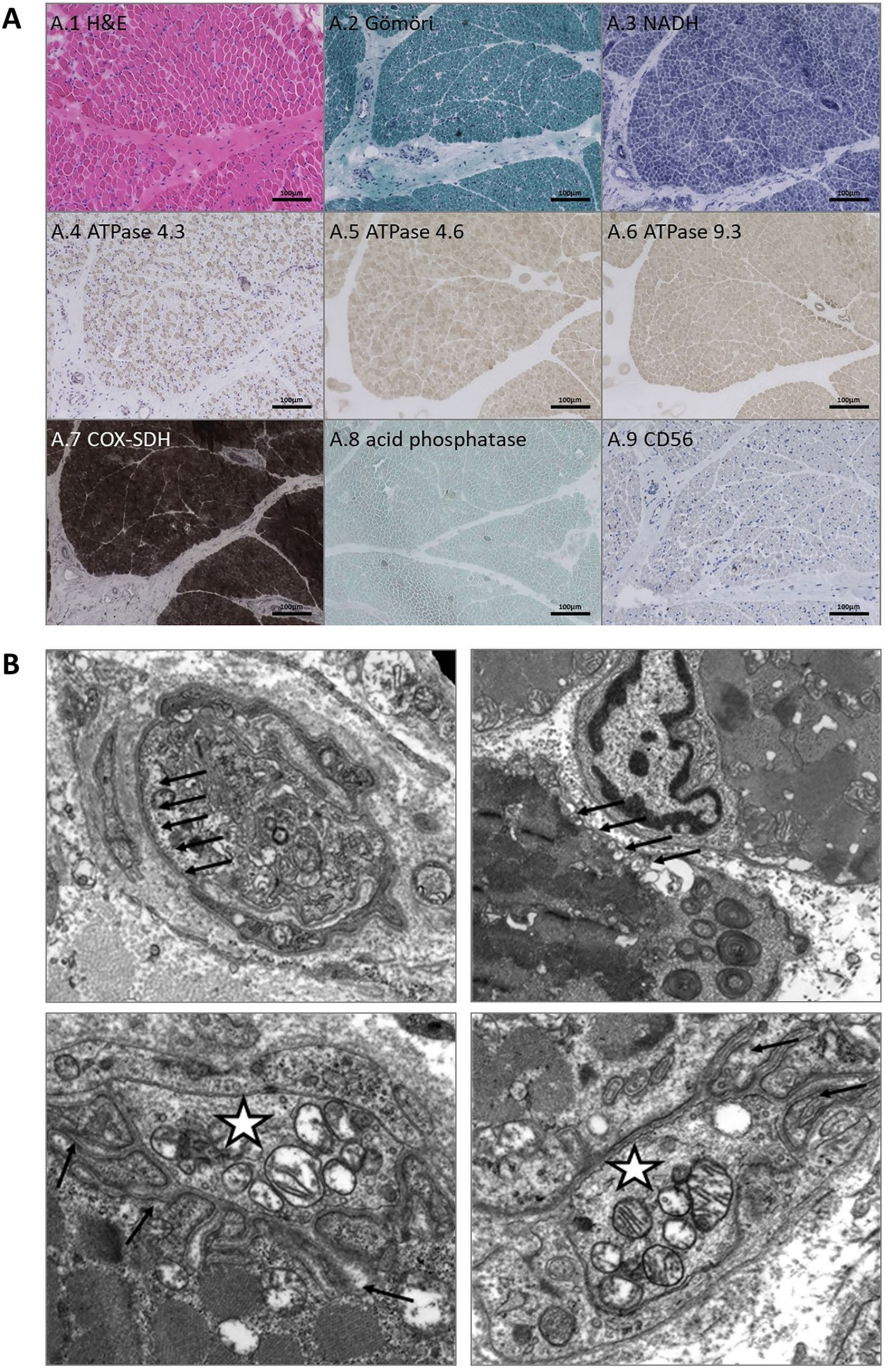
Microscopic analysis of quadriceps muscle biopsy from a 3-month-old *PURA* patient (P6). Light microscopy demonstrated largely preserved skeletal muscle architecture with a regular fiber caliber spectrum and no evidence of fiber-type grouping, fiber-type disproportion, inflammatory infiltrates, or structural abnormalities (**A1-8**). No signs of infantile neurogenic damage, mitochondrial alterations, or COX-negative fibers were observed. Electron microscopy revealed subtle and non-specific ultrastructural changes (**B1-4**). Endomysial capillary endothelial cells contained increased numbers of small vesicles aligned along the cell membrane, occasionally appearing to fuse with the basement membrane (**B1**). Subsarcolemmal vesicles of variable size and occasional cylindrical spirals were observed within myofibers (**B2**). Neuromuscular junctions showed presynaptic vesicles and mitochondria, with mild alterations of postsynaptic clefts, including partial widening and rarefaction (**B3-4**)

Microscopic studies were performed on a quadriceps muscle biopsy obtained from a 3-month-old *PURA* patient (P6; Fig. 1A, B) for diagnostic purposes prior to molecular genetic testing. Light microscopy revealed largely preserved muscle architecture with a regular fiber caliber spectrum and no evidence of fiber-type grouping, fiber-type disproportion, inflammatory changes, or structural abnormalities (Fig. 2A1-8). There were no signs of infantile neurogenic damage, mitochondrial pathology, or COX-negative fibers (Fig. 2A1-8), and membrane immunoreactivity for nNOS was age-appropriately absent (data not shown). Presence of motor end-point areas including NMJs were identified.

Electron microscopy demonstrated subtle and non-specific alterations, including mild changes in NMJ architecture with small postsynaptic clefts showing rarefication and partial widening. Endothelial cells displayed an increased number of small vesicles aligned along the cell membrane, occasionally appearing to fuse with the basement membrane (Fig. 2B1-4). Additional non-specific ultrastructural findings included cylindrical spirals, degenerated axons, loss of unmyelinated fibers, thickened vascular basal laminae, and partially swollen mitochondria (Fig. 2B1-4). Overall, these findings indicate subtle ultrastructural changes without definitive or disease-specific morphological hallmarks.

### Proteomic signature of *PURA*-mutant muscle

**Fig. 3:**
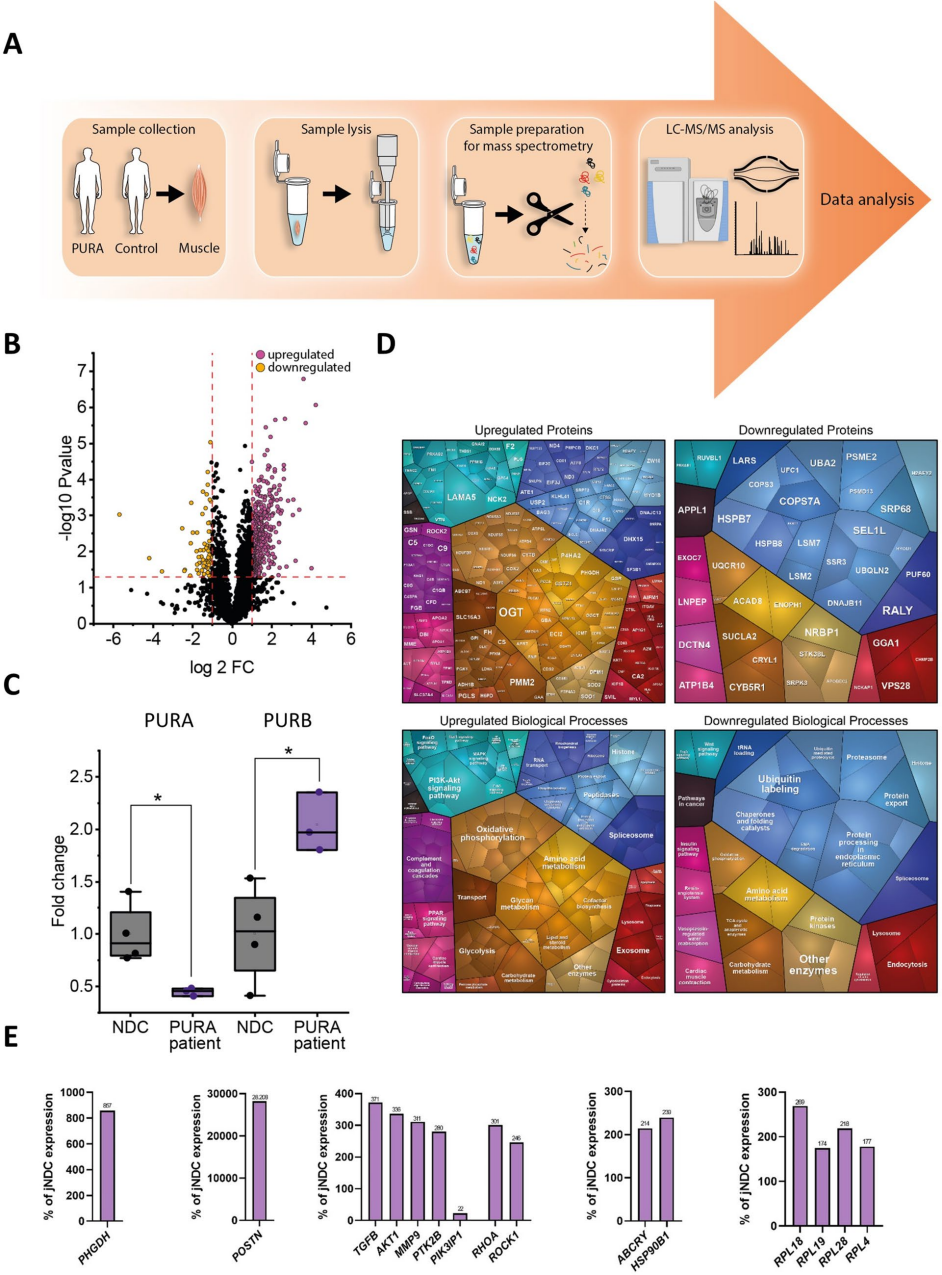
Proteomic profiling of quadriceps muscle derived from one *PURA* patient (P6) **A** Schematic representation of the applied workflow. **B** Volcano plot illustrates significantly upregulated (purple) and downregulated (yellow) proteins. **C** Box plot demonstrating reduced PURA and increased PURB protein abundances in patient quadriceps muscle compared to controls; patient quantification is based on technical triplicates. **D** Proteomaps-based in silico analysis of dysregulated proteins. The upper panels display protein-level alterations, while the lower panels highlight affected biological processes for upregulated (left) and downregulated (right) proteins. **E** Gene transcript analysis of periostin pathways and modulators of periostin expression in comparison to healthy juvenile controls demonstrates substantial increased expression. Additionally, based on the increase of ribosomal proteins in *PURA*-mutant muscle, transcript level of ribosomal proteins, showed an increase for all corresponding transcripts

Unbiased proteomic profiling making use of a DIA approach was conducted to elucidate the molecular fingerprint in the *quadriceps* muscle biopsy of P6 (Fig. 3A). Mass spectrometry-based protein quantification enabled the robust quantification of 28.693 unique peptides referring to 3.311 proteins. Of those, 431 were differentially abundant (13.02% of quantifiable proteins: 354 increased and 77 decreased) in muscle (Fig. 3B; Supplementary Table 1). In line with a heterozygous frameshift variant in *PURA* (p.(Leu54Cysfs*)), mass spectrometry-based protein analysis from muscle of P6 revealed a downregulation of the PURA protein (around 0.5-fold of controls; Fig. 3C; Supplementary Table 1). Notably, this was accompanied by an increase of PURB (more than twofold; Supplementary Table 1) in patient muscle compared to age- and gender-matched controls (*n* = 5) (Fig. 3C). Considering the established role of PURA in transcription, we manually filtered the dataset for proteins involved in transcriptional regulation, RNA processing, or chromatin remodeling, including transcription factors, co-factors, RNA-binding proteins regulating splicing, and chromatin-associated proteins. This analysis revealed an increase in 34 proteins and a decrease in 4 proteins related to these processes (Supplementary Table 1). Further manual filtering of data showed that approximately 10% of the upregulated and 13% of the downregulated proteins comprise COPI/COPII coat proteins, SNAREs, adaptors, Rab GTPases, tethering factors, cargo receptors, and other vesicle-associated proteins, all of which play essential roles in cellular vesicle transport. Of note, many of the increased proteins are localized to the extracellular matrix and are known to modulate fibrotic remodeling including POSTN (Supplementary Table 1).

Proteomaps term-based pathway analysis of the dysregulated proteins indicated, among others, an upregulation of diverse biological processes including complement activation, metabolisms (including oxidative phosphorylation, amino acid turnover, and glycolysis among others), splicing and histone function (indicative for heterochromatin remodeling), signaling cascades (such as PPAR, FoxO, MAPK and PI3K–Akt signaling), lysosome, as well as exosome functions (Fig. 3D). Downregulated proteins also impact on different signaling cascades (such as insulin and Wnt signaling), metabolic processes (oxidative phosphorylation, TCA cycle, and amino acid metabolism among others), splicing, tRNA loading, protein turnover (including protein processing in endoplasmic reticulum, ubiquitination, proteasome, as well as lysosome function), and endocytosis (Fig. 3D).

To complement proteomaps, GO-term enrichment analysis was performed to provide higher-resolution, statistically supported insights into the biological pathways and molecular functions affected by dysregulated proteins. This in silico approach confirmed the findings obtained by proteomaps, changes in heterochromatin remodeling, and in addition indicates that increased proteins impact on response to buildup of unfolded proteins as well as on sarcomere organization and, of note, synapse disassembly (Supplementary Fig. 1A). Along this line, GO term-based data analysis showed that decreased proteins also impact diverse biological processes including mRNA modification, membrane fission along with negative regulation of clathrin-dependent endocytosis, endosome transport via multivesicular body sorting pathway, retrograde vesicle transport to the Golgi, and various processes impacting on proteolysis including ubiquitin-dependent ERAD pathway, protein deneddylation, and regulation of macroautophagy (Supplementary Fig. 1A).

### Validation of the protein studies by qPCR

To validate paradigmatic protein findings and to additionally expand our muscle-derived proteomic findings to the gene level, we performed targeted analysis of transcripts encoding some of the dysregulated proteins. In line with an increase in protein level (more than eightfold; Supplementary Table 1), *POSTN* expression was expressed nearly 30.000 × more in comparison to healthy juvenile controls. In line with this considerable increase of *POSTN* expression, various transcripts for which the corresponding proteins are known modulators of periostin expression are also highly increased in patient’s muscle (Fig. 3E). *PHGDH* encoding D-3-phosphoglycerate dehydrogenase is 850-fold increased (Fig. 3E). Also, in line with increased protein level in *quadriceps* muscle, *ABCRY* and *HSP90B1* are 214- and 239-fold increased, respectively (Fig. 3D).

Based on the increase of ribosomal proteins in *PURA*-mutant muscle, transcript levels were studied for four ribosomal proteins, *RPL4*, *RPL18*, *RPL19,* and *RPL28*, showing an increase for all four corresponding transcripts between 170-and 270-fold (Fig. 3E).

### ELISA-based quantification of TSP4 in sera derived from *PURA* patients

**Fig. 4:**
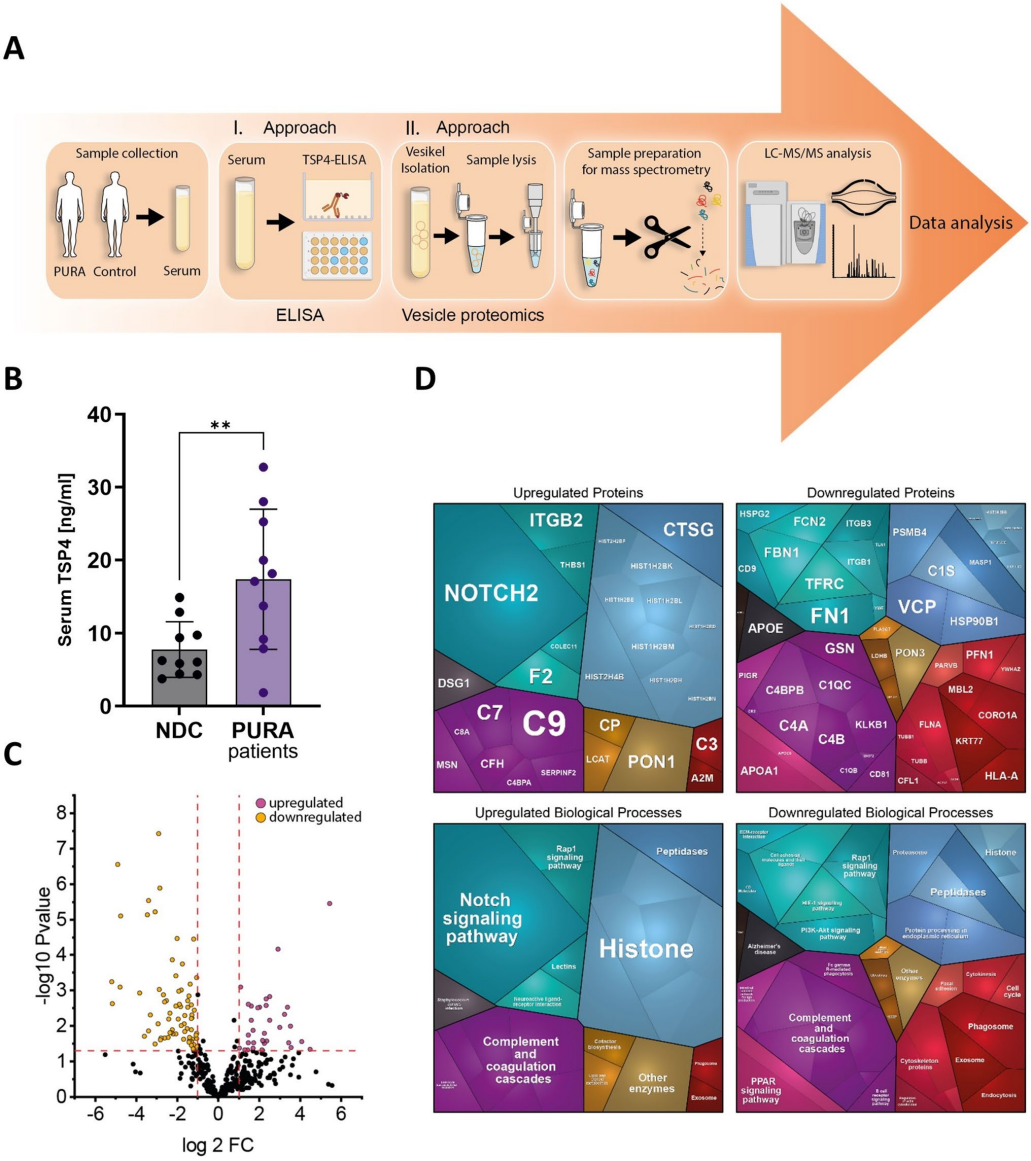
Protein studies on *PURA* patient blood samples toward biomarker identification **A** Schematic representation of the applied workflow. **B** Box plot shows the statistically significant increase of TSP4 in sera derived from *PURA* patients (average of 17.42 ng/ml in patient vs. 7.794 ng/ml in controls) whereby highest values were detected in P6. **C** Volcano plot illustrates significantly upregulated (purple) and downregulated (yellow) proteins in *PURA* patient derived sera compared to healthy pediatric controls. **D** Proteomaps-based in silico analysis of dysregulated proteins. The upper panels display protein-level alterations, while the lower panels highlight affected biological processes for upregulated (left) and downregulated (right) proteins

The investigation of TSP4 in the serum of *PURA* patients is based on results from one of our previous studies, in which we demonstrated that this protein, known to be localized at the NMJ [26], is altered in the cerebrospinal fluid of 5q-SMA patients. Moreover, the altered levels normalized following treatment with Nusinersen, a potent drug that also exerts a positive effect on neuromuscular transmission [29]. Serum TSP4 level quantified by ELISA (Fig. 4A) differs significantly (*p* = 0.0087) between *PURA* patients and healthy age-matched controls. Serum TSP4 was on average more than 2.3-fold more abundant in *PURA* patients than in the healthy controls (average of 17.42 ng/ml vs. 7.794 ng/ml) (Fig. 4B).

### Proteomic signature of EVs purified from *PURA* patient sera

Prompted by our proteomic and ultrastructural findings in muscle extracts, both indicating altered vesicle homeostasis, we next performed a proteomic discovery study on EVs purified from patient-derived sera (Fig. 4A). Mass spectrometry-based quantification revealed significant dysregulation of 126 proteins, with 44 increased and 82 decreased compared to controls (Fig. 4C; Supplementary Table 3). Among the upregulated proteins, NOTCH2 showed the strongest increase (43-fold), while PON1, previously implicated in muscle atrophy [31], was also markedly elevated (10.4-fold). Consistent with our findings in muscle extracts, several components of the complement system were dysregulated in EVs. In addition, 20 immunoglobulins exhibited altered abundance (11 increased, 9 decreased). Structural proteins were also affected, with 10 cytoskeletal proteins reduced, and 17 histone proteins significantly altered (10 increased, 7 decreased; Supplementary Table 3). Notably, TARSH, recently described as a circulating biomarker in CMS [30], was considerably decreased in patient-derived EVs (0.28-fold relative to controls). Interestingly, CD9 was also markedly reduced (0.27-fold relative to controls); this protein is known to inhibit myotube fusion during muscle regeneration (www.uniprot.org). A functional interplay of dysregulated proteins is illustrated in Supplementary Fig. 1B.

Proteomaps-based analysis of biological processes affected by dysregulated proteins revealed that vesicle-upregulated proteins primarily impact histone-related functions, as well as Notch signaling and activation of the complement system (Fig. 4D). In contrast, vesicle-downregulated proteins also contribute to complement cascade activation, modulate signaling pathways such as PPAR, Rap1, HIF-1, and PI3K-Akt, or are involved in protein folding and degradation. Notably, histones were also represented among the downregulated vesicular proteins **(**Fig. 4D).

## Discussion

Clinical, therapeutic, and experimental findings increasingly support a role for PURA in NMJ physiology, which fits with the observed clinical improvement of *PURA* patients treated with pyridostigmine, a cholinesterase inhibitor. However, the exact localization of PURA at the NMJ remains to be elucidated. Electrophysiological studies pointed to both pre- and postsynaptic localization [6]. Along this line, muscle biopsy findings have been reported in 15 cases thus far. In four cases, fiber-type disproportion (FTD) of skeletal muscle had been reported, varying from the diagnosis of congenital fiber-type disproportion to the mild, non-specific excess of type 1 fibers [3, 7]. Two cases showed type 2 fiber atrophy [32], and one had diffuse atrophy with limited details [3]. Myosin staining revealed increased neonatal myosin in one case [33] and mild fast fiber atrophy in another [32]. In H&E stains, muscle fibers occasionally showed central/internalized myonuclei [33]. In one case with clinical and electrophysiological presentation similar to that of a myasthenic syndrome, light microscopy showed increased muscle fiber size variability and smallness, but no pathognomonic features [33]. In three cases, skeletal muscles were described as normal [3]. Despite this prior knowledge, there are currently no robust, minimally invasive biomarkers to monitor NMJ defects, nor have ultrastructural NMJ abnormalities been described in *PURA* patients. Likewise, the biochemical basis underlying the muscle abnormalities remains unexplored. In this study, we combine proteomic, biomarker, and targeted morphological analyses to explore molecular alterations associated with *PURA*-related disease. While the structural and phenotypic assessments provide initial evidence of neuromuscular involvement, the principal contribution of our work lies in the identification of disease-associated proteomic signatures. Given the ubiquitous expression of *PURA*, these molecular changes cannot be attributed exclusively to neuromuscular synaptic dysfunction and may reflect broader cellular processes. Accordingly, our findings should be interpreted as highlighting candidate pathways and biomarkers relevant to disease pathophysiology rather than providing a definitive, integrated model of NMJ pathology.

On the clinical level, most of the symptoms present in our patients are like those described in other cohorts of *PURA* patients, such as neurodevelopmental delay, severe cognitive impairments, as well as muscle hypotonia [3, 4]. Hypotonia may partially reflect peripheral involvement, as deep tendon reflexes were reduced in two weak patients but increased in one. Further clinical features in line with perturbed neuromuscular transmission were ptosis, eye muscle weakness presenting as disconjugated gaze, as well as a myopathic facies. Of note, these symptoms have been noted in pediatric CMS cohorts [27]. Although most of the patients with mutations in established CMS genes (such as *DOK7, MUSK, RAPSYN, CHRNA1, CHRNB1, CHRND,* and *CHRNE*) do not show cognitive symptoms [34], in some newly identified CMS subtypes, myasthenia is only one element of a more severe and complex clinical spectrum [27]. This was recently exemplified for Noonan syndrome [35] and cognitive impairment has been documented in the context of the following conditions: *CHAT*-CMS, *LAMB2*-CMS, *SLC5A7*-CMS, *SNAP25*-CMS, *UNC13A*-CMS, *DPAGT1*-CMS, *ALG2*-CMS, *MYO9A*-CMS, and *SLC25A1*-CMS [8]. Our findings, together with clinical data from other studies, suggest that PURA-CMS may be considered within this group.

To further delineate the myopathological features associated with pathogenic *PURA* variants, we performed microscopic analyses of a quadriceps muscle obtained from one patient (P6) in our cohort. Light microscopy revealed no major structural or mitochondrial abnormalities and no evidence of fiber-type predominance or FTD, respectively. In contrast, electron microscopy demonstrated mildly swollen mitochondria, cylindrical spirals, vesicle accumulation, and alterations of NMJ architecture. These findings support the concept of NMJ pathology in *PURA* patients, and are in line with clinical and electrophysiological observations of impaired neuromuscular transmission and the therapeutic response to pyridostigmine. The results of our proteomic profiling approach on whole protein extracts of the *quadriceps* biopsy revealed reduced PURA protein levels, confirming the pathogenicity of the dominant *PURA* variant, and widespread dysregulation of proteins involved in transcriptional regulation, RNA processing, chromatin remodeling, and vesicle transport. Thus, while alterations in proteins involved in transcription, RNA processing, and chromatin remodeling are consistent with the established function of PURA (with PURB increase most likely in a compensatory manner), the dysregulation of proteins essential for vesicle transport aligns with our ultrastructural observations of putative vesicle accumulation and suggests that disturbed vesicle homeostasis may represent a relevant pathogenic mechanism in the etiology of this rare disease. Notably, several upregulated proteins localize to the extracellular matrix and are implicated in fibrotic remodeling, including POSTN. Fitting to this our targeted gene expression studies toward validation of paradigmatic proteomic findings revealed significant overexpression of several genes, notably *POSTN* and *PHGDH* confirming our proteomic findings on the gene expression level. Along with the increase of *POSTN*, we also identified a considerable increase of multiple factors (*TGFB, AKT1, MMP9, PTK2B, RHOA,* and *ROCK1*) known to activate *POSTN* expression. Notably, *POSTN* encodes periostin (POSTN), an extracellular matrix protein, which is not only known to modulate fibrotic remodeling in muscle [36], but also localizes within the synaptic clefts of NMJ in an intact muscle [37]. Several studies also reported muscular POSTN increase upon injuries, hinting toward a function also in muscle fiber regeneration [38]. Accordingly, it is difficult to attribute the upregulation of POSTN in the muscle of our *PURA* patient to any one of these functions. Since the skeletal muscle biopsy of our patient did not show fibrotic remodeling, as is typically seen in other genetic muscle disorders such as Duchenne muscular dystrophy (DMD), one could speculate that the increase in POSTN levels may rather be related to muscle regeneration or changes at the NMJ. However, further analyses, ideally in skeletal muscle from a mouse model, will be required to clarify this. *PHGDH* encodes phosphoglycerate dehydrogenase, an enzyme crucial for the serine metabolic pathway and muscle cell mass generation [39]. PHGDH inhibition decreased protein synthesis, myotube size, and myoblast proliferation through rapamycin complex 1 (mTORC1) [39]. Moreover, PHGDH is involved in L-serine-mediated function of synapses and was shown to represent a therapeutic target to restore NMJ and muscle pathology in *sil1*, *inpp5k,* and *phgdh* zebrafish morphants, resembling neuromuscular pathologies described in neuropediatric diseases caused by pathogenic variants in the respective human genes [40]. In summary, the upregulation of POSTN and PHGDH might be suggestive of regenerative or repair-related processes at the NMJ; however, definitive conclusions will require additional functional studies, particularly given the reliance on single-biopsy data. PIK3IP1 expression was reduced to approximately 20% of normal levels in skeletal muscle from P6. Although PIK3IP1 encodes a protein involved in the regulation of immunological signaling and has been implicated in autoimmune myasthenia gravis [41], this association most likely reflects a distinct pathogenic mechanism from CMS. Therefore, the observed reduction of PIK3IP1 in muscle of our PURA patient may point to altered regulatory or secondary signaling pathways rather than immune-mediated disease processes.

To explore minimally invasive biomarkers that might also provide insights into NMJ synaptic function, we analyzed serum concentrations of TSP4, a protein localized at the NMJ and recently identified as a marker of therapeutic response in the cerebrospinal fluid of patients with 5q-SMA [29]. Indeed, TSP4 levels were significantly increased in sera derived from *PURA* patients compared to gender- and age-matched controls, thus introducing TSP4 as a promising serum disease biomarker for PURA-CMS. Based on the known localization of TSP4 to the NMJ [26], we thus speculate that a forced TSP4 production may reflect an attempt to strengthen neuromuscular transmission under pathophysiological circumstances. Interestingly, the highest TSP4 level (32.8 ng/ml) was detected in the serum from our patient (P6), where muscle biopsy was performed and we described muscle pathology including structural NMJ changes. This finding lends further support to the hypothesis that elevated blood levels of TSP4 may reflect NMJ dysfunction. Nevertheless, Hoon Lee and colleagues reported histopathological evidence of chronic inflammatory changes in the cerebral arteries of *PURA* patients [42]. TSP4, a key regulator of vascular remodeling and extracellular matrix organization, has been implicated in neurovascular and inflammatory processes [43] and may therefore also contribute to such vascular pathology; accordingly, altered serum TSP4 levels in *PURA* patients may reflect vascular or neurovascular involvement rather than NMJ alterations alone, or potentially a combination of both mechanisms. Validation in larger cohorts, ideally incorporating neurophysiological assessments, will be necessary to substantiate the hypothesis of TSP4 serving as an NMJ biomarker and to better define the pathophysiological relevance of TSP4 as a minimally invasive biomarker in *PURA* patients. With regard to specificity, we acknowledge limitations in the use of serum TSP4 levels; although TSP4 is enriched at the NMJ [26], its expression in other tissues, including cardiac and vascular smooth muscle, may contribute to circulating levels.

In a further approach to identify minimally invasive biomarkers, we performed proteomic analyses of EVs isolated from the serum of our *PURA* patient cohort. This strategy was based on the hypothesis that vesicle pathology may contribute to the molecular pathology of PURA-CMS and on the established role of vesicles in synaptic information transfer [44]. Here, mass spectrometry-based protein quantification revealed significant dysregulation of multiple proteins, including immunoglobulins and components of the complement cascade. Interestingly, dysregulated histone proteins, detected within vesicles, might suggest a direct link between PURA’s role in gene regulation and the observed vesicle pathology, highlighting a novel molecular mechanism in *PURA* disease pathogenesis. Additionally, proteomic profiling revealed three interesting candidates in EVs of *PURA* patients. (i) NOTCH2, a protein which is necessary to internalize EVs and plays a neurotransmitter-like role [45]. At the NMJ, NOTCH2 is primarily abundant in the presynaptic cell bodies where its expression level is positively correlated with motor neuron activity [46]. The balanced level of Notch is necessary to provide a correct NMJ structure [46]. In muscle, NOTCH2 is necessary for the satellite muscle cells self-renewal [47]. This protein shows the highest increase in EVs derived from *PURA* patients. (ii) TARSH (decreased to 0.28-fold) has recently been identified in blood as a marker associated with *CHRNE*-related CMS [30]. Its levels thus may reflect NMJ dysfunction. However, given that the precise biological function of TARSH is not yet fully characterized, further studies are needed to clarify protein function, in particular in synaptic homeostasis. (iii) Although PON1 (10.4-fold increased) does not have a known direct role at the NMJ, its upregulation in muscle tissue or extracellular vesicles may reflect secondary responses to muscle stress or oxidative imbalance, which can indirectly affect NMJ integrity. PON1 is an antioxidant enzyme that hydrolyzes lipid peroxides, and elevated levels could indicate a compensatory mechanism in response to oxidative stress caused by dysfunctional synaptic activity and thus impaired neuromuscular transmission. In this context, increased PON1 in EVs (likely secreted by muscle cells) may serve as a biomarker of muscle vulnerability rather than a direct modulator of synaptic transmission, complementing other molecular signs of NMJ pathology observed in *PURA*-related disease. Along this line, PON1 was described as a potential novel atrokine for cancer cachexia and indicates localized inflammation in atrophying muscles [31]. Thus, although our results point toward a potential link between the identified biomarkers and synaptic dysfunction, the absence of neurophysiological validation limits definitive interpretation and highlights the need for future studies integrating functional assessments of the NMJ.

## Conclusions

Our data show that a pathogenic dominant *PURA* variant leads to reduced PURA protein levels in one muscle biopsy, associated with NMJ structural alterations, vesicle accumulation, and dysregulation of proteins involved in transcription, vesicle transport, and extracellular matrix remodeling. Clinical, histological, and molecular findings collectively support the current concept of a central role of PURA in NMJ physiology, consistent with the therapeutic response to pyridostigmine in *PURA* patients with perturbed neuromuscular transmission (*PURA*-CMS). Serum and extracellular vesicle analyses identify minimally invasive biomarker candidates, including TSP4, NOTCH2, TARSH, and PON1, which may reflect synaptic dysfunction and compensatory responses to muscle stress. Together, these findings may point toward a broader phenotypic spectrum in *PURA*-related disease, suggesting a potential association between cognitive impairment and myasthenic features, while underscoring the need for future studies to validate biomarker candidates and clarify disease mechanisms.

## Limitations

A major limitation of this study is the relatively small cohort size and the availability of only a single muscle biopsy per patient, which restricts longitudinal interpretation. Consequently, proteomic findings should be regarded as hypothesis-generating rather than definitive evidence of disease mechanisms. The absence of neurophysiological confirmation of NMJ dysfunction represents another major limitation, as such data would be necessary to more robustly link the observed biomarker changes to synaptic pathology; therefore, interpretations of these associations should be considered preliminary. Nevertheless, these findings may provide an important first indication and warrant validation in larger cohorts. In this context, it must be emphasized that in rare diseases, biomarker discovery is inherently challenging due to both the low prevalence of the disorder and the limited access to appropriate biomaterial for robust validation. This represents a general limitation in the neuromuscular field and is especially relevant for rare diseases such as *PURA*-related phenotypes.

With respect to the serum biomarker candidates identified, it should be noted that circulating biomarker levels may be influenced by confounding factors such as age, renal function, body mass index, and physical activity. Adjustment for these potential confounders was not performed in the present study but will be essential in future validation studies using larger patient cohorts.

## Supplementary Information

Below is the link to the electronic supplementary material.

Further results of in silico on proteomic data: (A) Bar plots resulting from GO-Term based studies of protein dysregulations in quadriceps muscle of P6 display significantly enriched biological processes associated with upregulated (left) and downregulated (right) proteins. The x-axis represents the adjusted p-value (FDR), while the y-axis lists the corresponding GO-terms. Upregulated proteins were primarily linked to complement activation, mitochondrial ATP synthesis, and immune responses as well as sarcomere organization and, of note, synapse disassembly among others. Downregulated proteins were associated with complement factors, RNA metabolism (mRNA modification, regulated splicing), protein folding and degradation (ERAD pathway, chaperone activity), and vesicle/endosomal transport. Together, these findings highlight distinct functional signatures for up- versus downregulated proteins in patient-derived vesicles. (B) STRING analysis of proteomic data obtained on serum EV of PURA patients shows functional clustering of dysregulated proteins into modules related to complement activation, lipid metabolism, cytoskeletal organization, histone function, and vesicle dynamics. The network highlights extensive interconnectivity, suggesting coordinated roles in neuromuscular and synaptic processes.
Supplementary file1 (JPG 898 KB)

List of proteins showing significant dysregulation in quadriceps muscle of one PURA patient (P6): The first sheet of the Excel table provides an overview of all statistically significantly dysregulated proteins. The second sheet lists proteins known to be involved in transcription. The third sheet summarizes proteins known to participate in vesicular transport.
Supplementary file2 (XLSX 56 KB)

Overview of the TaqMan probes used for targeted transcript quantification.
Supplementary file3 (XLSX 10 KB)

Overview of all proteins that are statistically significantly dysregulated in vesicles purified from serum of PURA patients.
Supplementary file4 (XLSX 23 KB)

## Data Availability

The proteomic datasets generated and analyzed during the current study are available from the ProteomeXchange Consortium via the [PRIDE] partner repository [48] under the accession number [PXD067750]. Other data that support the findings of this study are available on proper request from the corresponding author.
