# Supplementary figures and images for: Exploring molecular signatures in PURA syndrome using muscle proteomics and serum biomarkers
Source: J Neurol. 2026 Jan 23;273(2):94. doi: 10.1007/s00415-026-13621-7 (PMC12830457; doi:10.1007/s00415-026-13621-7)

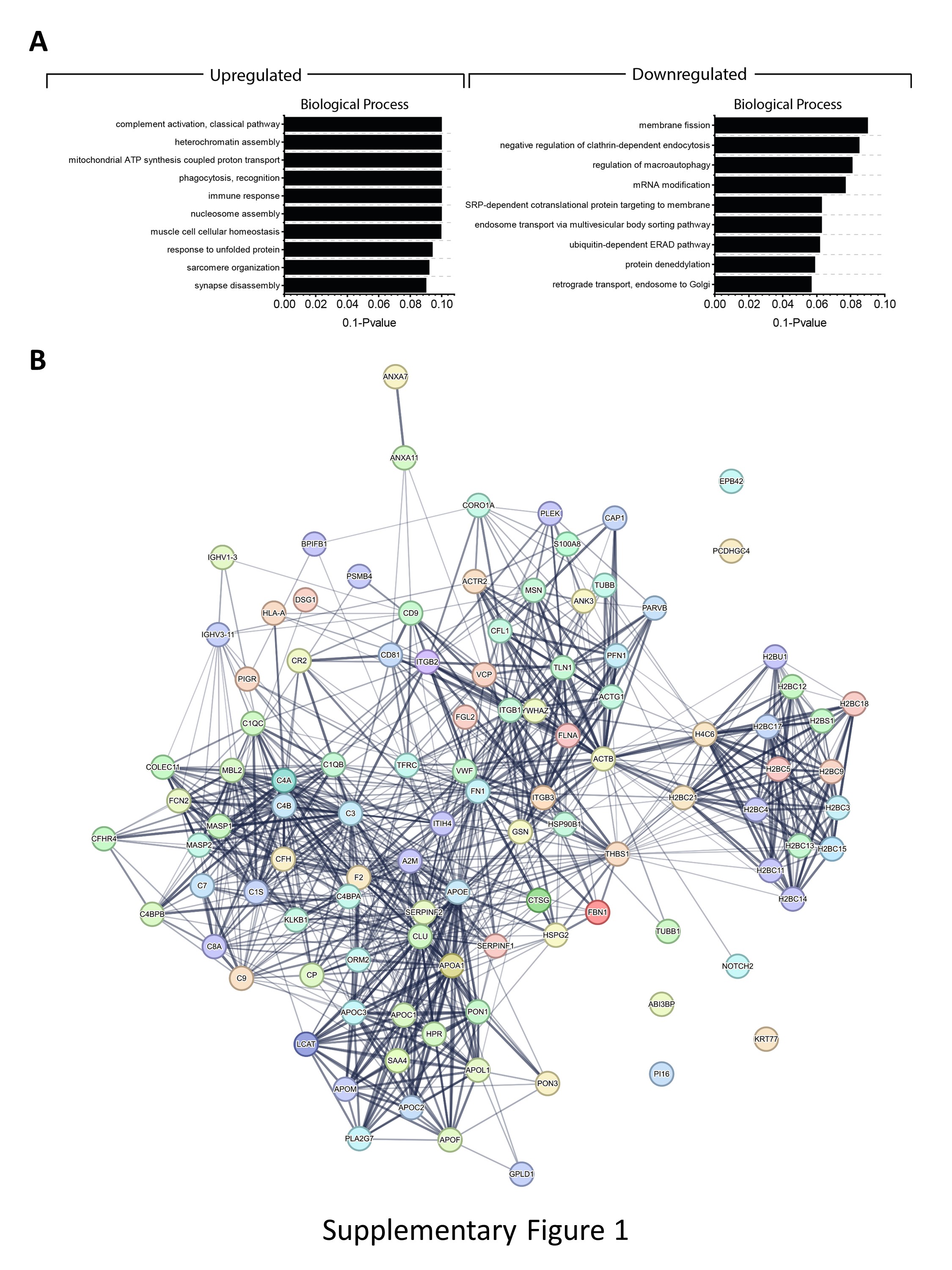

Supplement: Supplementary file 1 — Further results of in silico on proteomic data: (A) Bar plots resulting from GO-Term based studies of protein dysregulations in quadriceps muscle of P6 display significantly enriched biological processes associated with upregulated (left) and downregulated (right) proteins. The x-axis represents the adjusted p-value (FDR), while the y-axis lists the corresponding GO-terms. Upregulated proteins were primarily linked to complement activation, mitochondrial ATP synthesis, and immune responses as well as sarcomere organization and, of note, synapse disassembly among others. Downregulated proteins were associated with complement factors, RNA metabolism (mRNA modification, regulated splicing), protein folding and degradation (ERAD pathway, chaperone activity), and vesicle/endosomal transport. Together, these findings highlight distinct functional signatures for up- versus downregulated proteins in patient-derived vesicles. (B) STRING analysis of proteomic data obtained on serum EV of PURA patients shows functional clustering of dysregulated proteins into modules related to complement activation, lipid metabolism, cytoskeletal organization, histone function, and vesicle dynamics. The network highlights extensive interconnectivity, suggesting coordinated roles in neuromuscular and synaptic processes. Supplementary file1 (JPG 898 KB) [file 415_2026_13621_MOESM1_ESM.jpg]
